# Longitudinal tests on the influence of achievement goals on effort and intrinsic interest in the workplace

**DOI:** 10.1007/s11031-012-9318-1

**Published:** 2012-09-15

**Authors:** Ayumi Tanaka, Takuhiro Okuno, Hirotsugu Yamauchi

**Affiliations:** Faculty of Psychology, Doshisha University, 1-3 Miyakodani Tatara Kyotanabe, Kyoto, 610-0394 Japan

**Keywords:** Achievement goals, Effort, Intrinsic interest, Longitudinal study, Workplace

## Abstract

This study investigates whether four types of achievement goals—mastery-approach, mastery-avoidance, performance-approach, and performance-avoidance—influence effort and intrinsic interest at work. Cross-lagged panel analyses were applied to data from a two-wave survey conducted on 57 newly hired Japanese police officers. The results showed that performance-approach goals had significant positive influences on effort and intrinsic interest. In contrast, performance-avoidance goals had significant negative impacts on the abovementioned two outcome variables. Longitudinal effects were observed when the influence of competence expectancy was controlled. These results highlight the benefits of performance-approach goals and the costs associated with performance-avoidance goals in the workplace.

## Introduction

Studies have demonstrated that types of goals adopted when individuals confront achievement situations create different cognitive frameworks with regard to how one approaches and reacts to such situations (Dweck 1986; Nicholls 1984; Ames 1992). The achievement goal approach (Elliot 2005) has guided considerable research for over 30 years. In general, researchers (Ames and Archer 1988; Dweck and Leggett 1988; Nicholls 1984) have focused on two types of achievement goals—mastery and performance goals—that differ in terms of standards for defining and evaluating performance (Elliot and McGregor 2001; Urdan 1997). *Mastery goals* aim to develop and improve an individual’s skills and competence, which is in reference to past performances or an absolute standard. *Performance goals* aim to validate and demonstrate an individual’s competence compared to that of others; these goals adhere to a normative standard.

According to a more recent conceptualization of achievement goals, individuals exhibit either a positive (approaching success) or negative (avoiding failure) stance. Elliot (e.g., Elliot 1999; Elliot and McGregor 2001) and Pintrich (e.g., Pintrich 2000) included the concept of approach and avoidance dimensions to the mastery and performance goals (2 × 2 goals). This led to four goal classifications. *Mastery*-*approach goals* focus on the development of competence or the attainment of task mastery. *Mastery*-*avoidance goals* are defined by seeking to eschew a misunderstanding or failure to master a task. *Performance*-*approach goals* focus on better performance compared to others and attainment of favorable judgments of competence. Finally, *performance*-*avoidance goals* refer to the avoidance of inferior performance as compared to others, as well as unfavorable judgments of competence.

Several studies have suggested that achievement goals represent a potentially important construct in industrial and organizational research (e.g., Button et al. 1996; DeShon and Gillespie 2005). For an adult, work and organizational settings are important achievement situations. In terms of job engagement, the variety and complexity of task achievement, and its instrumentality to one’s life, differ greatly from those experienced in educational settings. However, the way that one views and approaches challenging situations, as well as how failure is handled, are as critical in the workplace as they are in the classroom. Compared to studies assessing achievement goals in an educational context, relatively few studies have examined them on a sample of adults in the workplace. Furthermore, with only a few exceptions (i.e., Chiaburu 2005; Chiaburu and Marinova 2005), very few studies provide in-depth comparisons among the four types of achievement goals. Therefore, the purpose of the present study was to examine whether 2 × 2 achievement goals affect an individual’s job pursuit. In addition, we sought to test the generalizability of evidence obtained from the educational domain.

We focused on two commonly studied behavioral and affective outcomes—effort and intrinsic interest. This was done because these two outcomes yield informative comparisons for evidence obtained from the academic domain (see Moller and Elliot 2006). As seen in the following sections, we build specific hypotheses based on the theoretical tradition of achievement goal research accumulated within the educational domain, and empirical evidence within the organizational settings, on the differential influence of four achievement goals on effort and intrinsic interest.

Individuals with mastery goals believe in the malleability of their abilities, and they focus on self-referenced improvement (Dweck 1999). These qualities are assumed to orient an individual toward valuing effort and resilience in the face of obstacles (Pintrich and Schunk 2002). VandeWalle et al. (1999) found that mastery goals were associated with sales performance through self-regulatory tactics. Therefore, mastery-approach goals ought to have a positive effect on effort. Although the precise role of mastery-avoidance goals has yet to be fully clarified, we expected that these goals would be characterized by perfectionist-like, continual, and self-referenced engagement as a way to avoid errors on the job (Elliot and McGregor 2001). Thus, mastery-avoidance goals should have a positive influence on effort.

Individuals with mastery-approach goals might derive enjoyment from their efforts when reaching their objectives, even when task requirements are high (Dweck 1986). Janssen and Van Yperen (2004) is one of the few studies that have examined the relationship between achievement goals and job-related affective outcomes; they observed a positive association between mastery-approach goals and job satisfaction. In contrast, Elliot (1999) suggests that for individuals with mastery-avoidance goals, self-regulation that is focused on potential negative outcomes leads to increased anxiety. Therefore, we expected that mastery-approach goals would have a positive influence on intrinsic interest, while mastery-avoidance goals would have a negative influence on intrinsic interest.

Individuals with performance-approach goals tend to exert a lot of effort in order to perform better than others (Van Yperen and Janssen 2002). Several studies (e.g., Elliot 1999; Pintrich 2000; Van Yperen and Janssen 2002) suggest that vulnerability to maladaptive helplessness response patterns—including low task effort and defensive strategies, both of which are typical of performance goals (Dweck 1999)—is more likely to be associated with performance-avoidance rather than performance-approach goals. For instance, Porath and Bateman (2006) reported a positive association between salespeople’s proactive behavior and performance-approach goals, as well as an association with mastery-approach goals. The authors also reported a negative relationship between performance-avoidance goals and both proactive behavior and job performance. Therefore, we expected a positive relationship with performance-approach goals, and a negative relationship with performance-avoidance goals, on effort.

Finally, positive affect should emerge so long as individuals with performance-approach goals seize the opportunity to display performance outcomes (Dweck 1986; see also Harris et al. 2005). However, when people are asked to perform new and complex tasks, they tend to be uncertain about their abilities to meet their competitive standards (Janssen and Van Yperen 2004). Therefore, we did not expect a relationship between performance-approach goals and intrinsic interest. In line with our hypothesis on mastery-avoidance goals, we expected there to be a negative relationship between performance-avoidance goals and intrinsic interest. This prediction follows from our assumption of the disruption of task involvement caused by anxiety under avoidance self-regulation.

To summarize, our research questions are related to the influence of job-related 2 × 2 achievement goals on effort and intrinsic interest in the workplace. Identifying these relationships might help supervisors or career educators better understand and enhance individuals’ behavioral and affective engagement within the workplace. This study expands on previously observed relationships through a longitudinal examination of achievement goals on effort and interest. Our cross-lagged panel design provides a better assessment of causal relationship. Additionally, individual differences in competence expectancy were used as covariates in our analyses. This technique is important in order to extract the unique effects of achievement goals, particularly performance-approach and performance-avoidance goals, as competence expectancy is often viewed as an antecedent to the manifestation of these goals (Elliot 1999).

## Methods

### Participants and procedures

Participants were newly hired police officers from a middle-sized prefecture located in central Japan (*N* = 57, 48 males and 9 females). The average age of the participants was 22.77 years (*SD* = 2.90). Participants were from two different employment categories: those who held a university degree (*N* = 34) and those who did not (*N* = 23).

Participants completed the same questionnaire at two different time points, one year apart. Similar to other newly hired police officers in Japan, participants were assigned to a police school for six to ten months for basic training. Participants first filled out the questionnaire four months after their entrance into this school (Time 1). After completing school training, participants underwent on-the-job training for seven to eight months. They then returned to the police school for an additional two to three months. It was during this time, after a final examination, that the participants filled out the questionnaire for the second time (Time2). One reason for the selection of this sample was a relatively homogeneous environment of participants, including living accommodations and salary.

### Measures

In order to avoid response bias related to central tendency for a Japanese sample (e.g., Si and Cullen 1998), all items pertaining to achievement goals, intrinsic interest, and competence expectancy were scored on a 6-point Likert-type scale, ranging from 1 (“not at all true of me”) to 6 (“very true of me”). Since scales on achievement goals, intrinsic interest, and competence expectancy were initially developed for use in educational settings, we modified the items slightly such that they were reflective of a work setting. Prior studies have confirmed the reliability and validity of these scales for Japanese student samples (Tanaka and Yamauchi 2000, 2001; Tanaka et al. 2006). Cronbach’s alphas for all scales used in the present study are provided in Table 1.

**Table 1:** Descriptive statistics of the variables (*N* = 57)

	Time 1		Time 2		*t*
	*M* (*SD*)	α	*M* (*SD*)	α	
Mastery-approach goals	5.27 (0.56)	0.74	5.09 (0.66)	0.83	2.52*
Mastery-avoidance goals	3.63 (1.19)	0.87	3.64 (1.04)	0.87	0.08
Performance-approach goals	3.41 (0.92)	0.83	3.01 (1.04)	0.90	3.15**
Performance-avoidance goals	2.78 (0.94)	0.67	2.64 (0.73)	0.51	1.05
Effort	3.85 (0.61)	0.66	3.81 (0.67)	0.67	0.44
Intrinsic interest	5.04 (0.79)	0.89	4.91 (0.71)	0.88	1.60
Competence expectancy	3.23 (0.89)	0.84	2.99 (0.82)	0.74	2.17*

* *p* < .05; ** *p* < .01

#### Achievement goals

The four types of achievement goals were assessed using the 12-item Achievement Goal Questionnaire (AGQ) developed by Elliot and McGregor (2001). In order to select items that were more suited to work settings, we added eight items from scales developed by Elliot and Church (1997) and Middleton and Midgley (1997). Responses to the 20 items for this scale were subjected to exploratory factor analyses using the principal factor method, which was followed by an oblique rotation. Four factors emerged with eigenvalues greater than 1, and each item was correlated with its appropriate factor. We retained 13 items that had high factor loadings on a priori factors and did not load on any other factor. Given that correlations between the factors were low, the final results, using a varimax rotation, are shown in the “Appendix”. Averaging the response created four subscales: mastery-approach, mastery-avoidance, performance-approach, and performance-avoidance goals. These results provide clear evidence that in our sample of Japanese adults, the 2 × 2 achievement goals are empirically distinguishable constructs in the work domain.

#### Effort

Effort was measured through three items taken from VandeWalle et al. (1999). Participants were required to rate time, work intensity, and overall effort devoted to their jobs in comparison to their coworkers. The items were translated from English to Japanese and then back translated. The response scale ranged from 1 (“much below the average”) to 6 (“much above the average”). Averaging the scores for the three items created the scale.

#### Intrinsic interest

Intrinsic interest in the job was assessed by five items, which were modified from a scale developed by Elliot and Church (1997) (e.g., “I am enjoying this job very much”). We excluded three items from the original scale because they were not applicable to the work domain. The scale was created by averaging the scores for the five items.

#### Competence expectancy

A two-item scale from Elliot and Church (1997) measured participants’ competence expectancy for the job. These items included, “I expect to do well in my job” and “I believe I will receive an excellent grade in my job.” Averaging the scores for the two items created the scale.

## Results

### Descriptive statistics

Tables 1 and 2 list descriptive statistics and correlations among our main variables. Gender was not correlated with any outcome variables, but age and employment group were significantly correlated with some of our outcome variables. Therefore, in subsequent analyses, we included age and dummy coded variables of the employment category and competence expectancy as control variables.

**Table 2:** Zero-order correlations of the variables (*N* = 57)

Variables	1	2	3	4	5	6	7	8	9	10	11	12	13
Time 1													
1. Mastery-approach goals	–												
2. Mastery-avoidance goals	−0.03	–											
3. Performance-approach goals	0.14	−0.05	–										
4. Performance-avoidance goals	−0.03	0.32*	0.23	–									
5. Effort	0.24	−0.27*	0.17	−0.18	–								
6. Intrinsic interest	0.50*	−0.10	0.03	−0.23	0.22	–							
7. Competence expectancy	0.21	−0.39*	0.48*	0.04	0.34*	0.07	–						
Time 2													
8. Mastery-approach goals	0.58*	0.01	0.07	−0.14	0.16	0.42*	0.08	–					
9. Mastery-avoidance goals	0.13	0.50*	−0.02	0.20	−0.01	−0.04	−0.13	0.28*	–				
10. Performance-approach goals	0.17	0.10	0.54*	0.19	0.23	0.01	0.27*	0.04	0.29*	–			
11. Performance-avoidance goals	0.13	0.32*	0.23	0.35*	0.02	0.02	0.14	0.11	0.53*	0.41*	–		
12. Effort	0.04	−0.07	0.30*	−0.13	0.41*	0.05	0.30*	0.22	0.06	0.31*	0.08	–	
13. Intrinsic interest	0.34*	0.07	0.16	−0.29*	0.13	0.67*	−0.03	0.53*	0.10	0.02	−0.13	0.17	–
14. Competence expectancy	0.11	−0.15	0.48*	−0.15	0.20	0.04	0.54*	0.06	−0.18	0.46*	0.11	0.49*	0.02

* *p* < .05

Table 1 shows that the participants had significantly lower mastery-approach and performance-approach goals and competence expectancy at Time 2 as compared to Time 1. Table 2 shows a significant correlation between the same constructs across the two waves (Time 1 and Time 2), indicating some level of stability within each construct over time. Table 2 also shows a positive correlation between mastery-approach goals and intrinsic interest both at Time 1 and Time 2, a negative correlation between mastery-avoidance goals and effort at Time1, and a positive correlation between performance-approach goals and effort at Time 2.

### Longitudinal impact of achievement goals

Hierarchical regression analyses were used to test the longitudinal impact of achievement goals on effort and intrinsic interest. Step 1 of the analyses included effort and interest at Time 1 and control variables (competence expectancy, age, and employment group). Step 2 included all four achievement goals at Time 1. The results are shown in Table 3.

**Table 3:** Regression analyses predicting changes in outcome variables

Predictors (Time 1)	Outcome variables (Time 2)			
	Effort		Intrinsic interest	
	Step 1	Step 2	Step 1	Step 2
Step 1				
(Outcome variables)				
Effort	0.36**	0.33*	–	–
Intrinsic Interest	–	–	0.62***	0.62***
(Control variables)				
Competence Expectancy	0.11	0.03	−0.11	−0.11
Age	0.15	0.22	−0.07	−0.07
Employment category	−0.12	−0.21	−0.03	−0.03
Step 2				
(Achievement goals)				
Mastery-approach goals	–	−0.09	–	0.01
Mastery-avoidance goals	–	0.19	–	0.16
Performance-approach goals	–	0.34*	–	0.26*
Performance-avoidance goals	–	−0.32*	–	−0.25*
Δ*R* ^2^	0.25	0.13	0.48	0.08
Δ*F*	4.41**	2.60*	11.85***	2.22^†^

The coefficients are the beta weights

^†^ *p* < .10; * *p* < .05; ** *p* < .01; *** *p* < .001

The results in Step 2 show that achievement goals at Time 1 accounted for 13 % of the variance in effort at Time 2, *F* (4, 48) = 2.60, *p* < .05, over and above 25 % of the variance accounted for by effort and control variables at Time 1. The performance-approach goals were significant positive predictors of the residual effort scores at Time 2 (*β* = 0.34, *p* < .05), whereas the performance-avoidance goals were negative predictors (*β* = −0.32, *p* < .05). The mastery-approach and avoidance goals were not significant predictors of the residual effort score.

The results in Step 2 also show that achievement goals at Time 1 accounted for 8 % of the variance in intrinsic interest at Time 2, *F* (4, 48) = 2.22, *p* < .10, over and above 48 % of the variance accounted for by interest and control variables at Time 1. There was a substantive auto-regression effect (*β* = 0.62, *p* < .001) indicating that intrinsic interest remained stable across time. Further, performance-approach goals were significant positive predictors of the residual intrinsic interest scores at Time 2 (*β* = 0.26, *p* < .05), whereas the performance-avoidance goals were negative predictors (*β* = −0.25, *p* < .05). Here again, the mastery-approach and avoidance goals were not significant predictors of the residual intrinsic interest score.

To test for reverse causation, we ran an additional set of regression analyses. Each of the four achievement goals at Time 2 was regressed on itself, with the outcome variables (effort and intrinsic interest) and the control variables, at Time 1. As shown in Table 4, none of the outcome variables at Time 1 predicted residual achievement goal scores; thus, it appears that achievement goals influenced outcome variables rather than the reverse.

**Table 4:** Regression analyses predicting changes in achievement goals by outcome variables

Predictors (Time 1)	Achievement goals (Time 2)			
	Mastery-approach	Mastery-avoidance	Performance-approach	Performance-avoidance
Mastery-approach goals	0.49***	–	–	–
Mastery-avoidance goals	–	0.56***	–	–
Performance-approach goals	–	–	0.56**	–
Performance-avoidance goals	–	–	–	0.34*
Effort	0.03	0.11	0.15	0.05
Intrinsic interest	0.20	0.08	0.02	0.12
Competence expectancy	−0.09	−0.03	−0.10	0.01
Age	0.14	0.17	0.10	0.37*
Employment category	0.00	−0.15	−0.11	0.12
*R* ^2^	0.37***	0.34**	0.34**	0.24*

The coefficients are the beta weights

* *p* < .05; ** *p* < .01; *** *p* < .001

## Discussion

The current study aimed to extend the 2 × 2 achievement goal approach to the workplace domain and investigate whether and how achievement goals predict motivational outcomes (i.e. effort and intrinsic interest) over time. Based on the theoretical tradition of achievement goal research in the educational domain, and empirical evidence within other organizational settings, we expected there to be a positive relationship between mastery-approach goals on both job-related effort and interest in the job. We also predicted a positive relationship between mastery-avoidance goals and effort and a negative relationship between these goals and interest. Additionally, we expected to observe a positive relationship between performance-approach goals and effort and a null relationship between these goals and interest. Finally, we predicted a negative relationship between performance-avoidance goals and both effort and interest. To test our hypotheses, a longitudinal survey was conducted on a sample of newly hired police officers.

Our hypotheses were partially supported. The results from an exploratory factor analysis for the achievement goal items demonstrated a clear separation of a 2 × 2 achievement goal structure within the workplace. The results from our hierarchical regression analyses indicated that four types of achievement goals adopted several months after starting a career differentially predicted changes in effort and intrinsic interest after one year.

First, contrary to our hypotheses we did not find any hypothesized influence of mastery-approach or mastery-avoidance goals on effort and intrinsic interest. A long-standing assumption in achievement goal research is that mastery goals are superior to performance goals in many aspects, including greater task effort and interest (Payne et al. 2007). While our cross-sectional data in Table 2 did observe some significant correlations, there was no influence of mastery-approach goals, as well as mastery-avoidance goals, on longitudinal change in effort or intrinsic interest. Second, in line with our predictions, individuals with performance-approach goals showed an increase in effort, and individuals who adopted performance-avoidance goals showed a decline in both effort and interest in the workplace, independent of an individual’s competence expectancy. Although we expected a null relationship between performance-approach goals and interest, performance-approach goals positively predicted interest one year later. The present results offer support for the notion that performance-approach goals often produce greater engagement and performance in a task than do mastery-approach goals (see Elliot and Moller 2003; Harackiewicz et al. 2002).

One possible explanation for the lack of a significant influence of mastery-approach goals and the overall positive effects of performance-approach goals is low job autonomy and difficulty developing self-referenced competence in the working environment among the current sample. Police duties are associated with higher-level rules and regulations (Saikawa 2002). In the current study, both approach goals (mastery-approach and performance-approach) and competence expectancy were significantly lower at Time 2 compared to at Time 1. This could imply that for newly hired workers, most of the tasks might be new and difficult to approach. In a comparison between mastery-approach goals and performance-approach goals, Senko et al. (2011) argued that individuals with the former are freer to pursue their own learning agenda and guided by their curiosity and personal interests. Pursuing mastery-approach goals and striving to gain competence based on self-defined criteria might not be optimal for job performance in the current sample. In contrast, Vermetten et al. (2001) found that performance-focused students are particularly attentive to instructors’ demands and reliant on instructors’ cues on how to approach their studies. It is possible that in the early stages of an individual’s career, adopting performance-approach goals and striving for normative competence is more functional and facilitative than adopting mastery-approach goals. Future industrial and organizational research should examine the changes in achievement goal functions at different career stages.

While the current study offers important insights into the interrelationships between achievement goals and work-related behaviors, it does have several limitations worth mentioning. One important limitation concerns the size^1^ and specific nature of our police sample. Although this sample allows us to control for working conditions, including job autonomy, accommodation, and salary, it also undoubtedly limits the generalizability of the present results to different workplaces. Therefore, future studies should address the interactive effect of achievement goals and the perceived work environment on organizational behavior (see Chen and Mathieu 2008).

Another limitation is that the items used to measure 2 × 2 achievement goals were a combination of several scales. Although our scale items do capture the construct accurately, and the correlational analysis did show substantial discriminant validity, it is important to use a more common and refined measure to further enhance the generalizability of the findings. In their review of achievement goal literature, Elliot and Murayama (2008) identified several problems with existing measures, including the Achievement Goal Questionnaire (AGQ) (Elliot and McGregor 2001). For example, they pointed out that some items in the AGQ suggested a value (e.g., “It is important for me to do better than other students”) or a concern (e.g., “I worry that I may not learn all that I possibly could in this class”) rather than a goal, per se. Therefore, forthcoming studies will benefit from the application of revised achievement goal scales (Achievement Goal Questionnaire—Revised: AGQ-R) (Elliot and Murayama 2008) within the work domain. Further, Elliot and Murayama (2011) recently proposed a new dimension to the definition of competence (task-based, self-based, and other-based); this new dimension is potentially suited to organizational settings as well.

As a final limitation, our study employed only self-reported data and did not assess job performance, which is likely a major concern for organizations. This should be included in future investigations. Future research will need to examine how the interaction between achievement goals and actual performance can result in changes in effort and interest.

Despite the limitations of the present study, our findings contribute to a greater understanding of the motivational factors that foster engagement in the workplace. We demonstrated that higher performance-approach goals and lower performance-avoidance goals might be an important positive and negative source of effort and intrinsic interest in the workplace. Supervisors and/or mentors should first be aware of the importance of individuals’ achievement goals for an active and productive work life of their employees. Finally, with a few exceptions (e.g., Daniels et al. 2009), the longitudinal predictability of achievement goals has not been fully investigated. Further longitudinal research is necessary to accurately examine the impact of achievement goals.

## Appendix

See Table 5.

**Table 5:** Factor structure of the achievement goal questionnaire (*N* = 57)

Items	Factor			
	1	2	3	4
Mastery-approach				
I want to learn all that there is to learn in the job	0.07	**0.83**	0.02	−0.15
I hope to gain a broader and deeper knowledge of the job	−0.01	**0.69**	0.07	0.13
I want to learn as much as possible from the job	0.01	**0.59**	−0.04	−0.05
I desire to completely master the task presented in the job	−0.04	**0.52**	0.18	−0.07
Mastery-avoidance				
I am often concerned that I may not learn all that there is to learn in the job	**0.89**	0.02	−0.05	0.18
I worry that I may not learn all that I possibly could in the job.	**0.83**	0.03	0.04	0.02
Sometimes I’m afraid that I may not understand the content of the job as thoroughly as I’d like	**0.74**	−0.03	−0.05	0.15
Performance-approach				
My goal in the job is to get a better result than most of the other workers	0.01	0.12	**0.83**	0.20
It is important for me to do better than my coworkers	−0.04	0.14	**0.78**	−0.02
It is important for me to do well compared to others in the job	−0.01	−0.04	**0.73**	0.14
Performance-avoidance				
My goal in the job is to avoid performing poorly	0.03	0.31	0.11	**0.84**
I do my job so others in my workplace won’t think I’m dumb	0.25	−0.25	0.09	**0.59**
My fear of performing poorly in the job is often what motivates me	0.12	−0.18	0.12	**0.56**
Percentage of variance explained	16.38	15.46	14.77	11.77

Loadings greater than 0.50 are given in boldface

## Footnotes

1 In the present study, we conducted power analyses using Power and Precision 4.1 (Borenstein et al. 2001). The analyses estimated that at power = 0.80 and *p* = .05, a study with a sample size of 57 could detect a population increment *R* ^2^ of 0.15 by a set of four variables, over and above a set of four covariates with population *R* ^2^ of 0.25 (=Step 1 increment for Time 2 effort in the present results). Based on the power analyses, it can also be estimated that if the population *R* ^2^ increment by a set of four variables is 0.08 over and above the set of four covariates with *R* ^2^ of 0.48 (=the present results for intrinsic interest at Time 2), then the power is 0.65 with the present sample size of 57 at *p* = .05.
